# Priorities for the Priority Review Voucher

**DOI:** 10.4269/ajtmh.16-0600

**Published:** 2017-01-11

**Authors:** David B. Ridley

**Affiliations:** 1 Duke University, Durham, North Carolina.

## Abstract

The U.S. Congress created the priority review voucher program in 2007 to encourage development of drugs for neglected diseases. Under the voucher program, the developer of a drug for a neglected or rare pediatric disease that is approved by the U.S. Food and Drug Administration receives a bonus priority review voucher for another drug. As of 2016, four vouchers have sold for an average price of $200 million. Recent experience with the voucher program indicates strengths and weaknesses of the program, as well as a need for legislative changes.

The U.S. Congress created the priority review voucher program in 2007 to encourage development of drugs for neglected diseases. Congress expanded the program in 2012 to include rare pediatric diseases. Under the voucher program, the developer of a drug for a neglected or rare pediatric disease that is approved by the U.S. Food and Drug Administration (FDA) receives a bonus priority review voucher for another drug. Thus, two drugs are involved: the drug that wins a bonus priority review and the drug that uses the bonus priority review. The drugs need not be from the same company, because the voucher may be sold. Priority review typically reduces review time at the FDA by about 4 months.^1^,^2^ As of June 2016, 10 vouchers had been awarded and four had been sold for an average price of around $200 million.^3^

Based on their first-hand experience with the voucher program, industry executives Berman and Radhakrishna evaluate the priority review voucher program in a recent paper in this journal.^4^ They highlight perceived flaws in the voucher program that could reduce expected returns to drug developers.

First, Berman and Radhakrishna argue that incentives for drug development created by the voucher program are limited by the narrowness and uncertainty of voucher eligibility. To be eligible to receive a bonus priority review, a developer's drug must itself receive priority review. In other words, there are two drugs with priority review for every voucher. To be awarded a voucher, the drug for a neglected or rare pediatric disease must earn priority review on its own merit by offering a major advance in treatment, or providing treatment where no adequate therapy exists. If a competing drug wins a race to market, then subsequent drugs for that disease are less likely to qualify for a voucher. The developer does not know prior to submission whether it will receive priority review. For a marginal drug, the uncertainty could discourage investment. Another critique of the eligibility criteria is that previously approved molecules are ineligible, even if they target new indications.

Fortunately, developers can receive signals from FDA about the likelihood of receiving priority review. Drugs with accelerated approval or fast track status are highly likely to receive priority review. Also, for drugs treating rare pediatric diseases, the FDA provides a rare pediatric disease designation early in the development process. Furthermore, while the eligibility criteria could reduce investment in marginal drugs, it could also increase investment in innovative drugs. Because the voucher program is subject to supply and demand, reducing the quantity of vouchers pushes prices higher, which encourages development.^2^ Indeed, Congress could tighten eligibility even more. For drugs already available outside the United States, Congress could require that the manufacturer certify that it conducted new clinical investigation after the 2007 law.

Second, Berman and Radhakrishna argue that clinical trial costs are higher for drugs for neglected diseases than for rare, pediatric diseases, because developers of drugs for rare diseases are often permitted by FDA to conduct smaller trials. However, the cost of testing for neglected diseases such as dengue, malaria, and tuberculosis might not be so onerous relative to other drugs because of the ease of identifying patients for a clinical trial. Also, drugs with greater clinical benefits require smaller trials to prove that the benefits are real. Indeed, there is evidence that drugs for infectious and parasitic diseases tend to have lower development costs than other drugs.^5^

Third, Berman and Radhakrishna argue that expected returns are reduced by legislative risk. Investors cannot be certain that a drug will win a voucher because they do not know how eligibility might evolve. Indeed, there is some risk that the rare pediatric program will not be renewed, although renewal seems likely given the affinity for the voucher program by members of Congress.

In support of the voucher program, Berman and Radhakrishna argue that the voucher program imposes little cost to society or to other products under FDA review. This is a goal of the program and the reason that the voucher user must pay an additional FDA user fee of $2.7 million (in addition to the usual $2.4 million user fee for a total of $5.1 million). However, even with the additional user fee, FDA cannot easily hire new staff each time a voucher is redeemed. FDA needs a steady stream of vouchers to be confident that it can commit to hiring staff. Furthermore, FDA struggles to attract new staff given Federal employment and pay limits.^6^ Congress should loosen FDA pay limits. Also, if several vouchers were redeemed every year, FDA could confidently hire many new staff. For example, if four vouchers were redeemed each year, then FDA would receive an extra $11 million each year that could be used to hire around 22 new reviewers.

Finally, although the voucher program encourages drug development, the program does not ensure access to the drugs.^1^,^2^,^7^,^8^ Congress should require that manufacturers have a plan for ensuring drug access, especially for drugs for neglected diseases. This would help motivate developers to work with international organizations to promote access. Furthermore, some international organizations might support drug access through funding such as an advance market commitment.^9^

The voucher program would benefit from refinement. It is helpful to have policy makers, regulators, public health-care advocates, academics, and manufacturers engaged in a debate about the strengths and weaknesses of the voucher program to enhance the program for the future.
